# Complete genome sequences of two polyvinyl-alcohol-degrading bacteria

**DOI:** 10.1128/mra.00350-25

**Published:** 2025-06-09

**Authors:** Kohei Nakamura, Furawa Suzuki, Masashi Nambu, Nozomi Kawada, Atsushi Tanikawa, Nobuaki Naganuma

**Affiliations:** 1 Faculty of Applied Biological Sciences, Gifu University 12785, Gifu, Japan; 2 Basic Technology Research Division R&D Department, AICELLO CORPORATION 687542 , Toyohashi, Aichi Prefecture, Japan; University of Southern California, Los Angeles, California, USA

**Keywords:** polymers, biodegradation, polyvinyl alcohol

## Abstract

In this study, we report the complete genome sequences of two polyvinyl alcohol (PVA)-degrading strains: *Rhodanobacter* sp. DHB23 and *Flexivirga endophytica* CGMCC 1.15085. Their genome lengths were estimated to be 4,019,238 bp and 4,495,174 bp, respectively.

## ANNOUNCEMENT

The biodegradation processes of polyvinyl alcohol (PVA) have been reported since the 1970s (1). PVA or vinyl alcohol oligomers are initially oxidized by PVA dehydrogenase (PVADH) and/or PVA oxidase or secondary alcohol dehydrogenase (SADH), and the resulting oxidized PVA is subsequently hydrolyzed by oxidized PVA hydrolase (OPH) (1). The genome sequencing of a PVA degrader, *Stenotrophomonas rhizophila* QL-P4, has recently led to the discovery of a novel PVA oxidase (2). Here, we report the genome sequences of two PVA decomposers.

Activated sludge from the Ogaki City municipal wastewater treatment plant was sampled and introduced to an enrichment of isolated PVA degraders that was developed in our previous study (3). Measurement of the PVA (SOLUBLONGA, Aicello CO LTD, Japan) concentration within the culture supernatant was performed via the iodine-borate method (4). We purified strains G1 and G2 from each single colony developed on the PVA agar and confirmed that each strain formed clear zones on iodine-stained PVA plates.

We collected cells from strains G1 and G2 grown on PVA agar at 30°C for 4 days and extracted DNAs using DNeasy Blood & Tissue Kit (QIAGEN). The extracted DNAs without shearing and size selection were used for both DNA sequencings using Illumina MiSeq and Oxford Nanopore MinION platforms. MiSeq and MinION sequencings were performed with Reagent Kit v3 and 2 × 300 bp paired-end reads using Illumina DNA Prep Kit and IDT for Illumina Nextera DNA CD Indexes and with R10.4.1 flow cell and Rapid Barcoding kit, respectively. We obtained 1,295,224 and 883,261 reads from MiSeq and 419,393 and 72,855 reads from MinION for strains G1 and G2, respectively.

We used the default parameters for all software processing unless otherwise stated. We trimmed and filtered the raw reads for quality, sequencing adapters, and length using fastp v.0.23.4 (5) for MiSeq and dorado (v.0.7.2 https://github.com/nanoporetech/dorado) with superaccurate base-calling model, Porechop (v.0.2.4 https://github.com/rrwick/Porechop), and Filtlong (v.0.2.1 https://github.com/rrwick/Filtlong) for MinION. The both quality-controlled reads were assembled using Unicycler v.0.5.1 (6). Assembly yielded one closed contig (4,019,238 bp) and three closed contigs (4,495,174, 166,025, and 63,733 bp) for strains G1 and G2, respectively. *De novo* assembled graphs were visualized using Bandage v.0.8.1 to confirm the circularities of the contigs (7). The contigs were computed using QUAST v.5.0.2 (8) for genome statistics (Table 1). Sequences were rotated and annotated using Prodigal v.2.6.3 (9), ARAGORN v.1.2.38 (10), CRT v.1.2 (11), and barrnap v.0.8 (12) in online DFAST (13). Quality control and classification of chromosomal sequences were conducted using Checkm2 v.1.0.2 (14) and GTDB-Tk v.2.4.0 (15). Plasmid sequences were identified with Plasmer v.0.1 (16). The DDBJ/ENA/GenBank accession numbers are shown in Table 1.

**TABLE 1:** Sequencing metrics and genome statistics

Strain	G1	G2		
	Chromosomal	Chromosomal	Plasmid 1	Plasmid 2
Raw sequence reads				
Illumina paired-end read length (nucleotides)	300	300		
Number of Illumina reads	1,295,224	883,261		
Number of Nanopore reads	419,393	72,855		
Nanopore read N50 (bp)	7,793	13,730		
Assembly statistics for closed sequences				
Total sequence length (bp)	4,019,238	4,495,174		
Number of sequences	1	1		
GC content (%)	68	68		
Number of CDSs	3,464	4,283		
Coding ratio (%)	89	92		
Number of rRNAs	6	6		
Number of tRNAs	60	50		
Number of CRISPRS	1	-		
Average Illumina coverage (×)	81	57		
Average Nanopore coverage (×)	174	107		
CheckM2 result				
completeness and contamination (%)	99.99, 0.1	100.0, 0.47	NA^*a*^	NA
Taxonomy assignment				
GTDB Taxonomy (accession no., ANI^*b*^ %)	Rhodanobacter sp. (GCF_014842975.1, 89.54)	Flexivirga endophytica (GCF_014640635.1, 83.62)	NA	NA
Locus numbers of homologous genes related to PVA degradation^*c*^				
PVA dehydrogenase (PVADH)	RBSG1_17540	ND^*d*^	ND	ND
Secondary alcohol dehydrogenase (SADH)	RBSG1_20630, RBSG1_22550, RBSG1_23660	FVSG2_01440, FVSG2_02440, FVSG2_12740	ND	ND
Oxidized PVA hydrolase (OPH)	RBSG1_31750	FVSG2_13950	ND	ND
GenBank accession no.				
BioProject accession no.	PRJDB20270	PRJDB20272		
Sequence Read Archive no. (Illumina)	DRR640539	DRR640541		
Sequence Read Archive no. (Nanopore)	DRR640540	DRR640541		
BioSample accession no.	SAMD00886145	SAMD00886146		
Accession no.	AP039674	AP039675	AP039676	AP039677

^*a*^ Not applicable.

^*b*^ Average nucleotide identities.

^*c*^ PVA oxidase was not found in any sequences.

^*d*^ Not detected.

One chromosomal DNA was retrieved from strain G1, while one chromosomal and two plasmid DNAs from strain G2. Genome sequence similarity analysis showed that strains G1 and G2 were related to *Rhodanobacter* sp. DHB23 and *Flexivirga endophytica* CGMCC 1.15085, respectively (Table 1). Homologs of PVADH, SADH, and OH were found in strain G1, while those of SADH and OPH in strain G2 (Table 1). PVADH and PVA oxidase were not found in strain G2; therefore, its PVA-degrading mechanism remains to be clarified.
